# A Systematic Study on Electromyography-Based Hand Gesture Recognition for Assistive Robots Using Deep Learning and Machine Learning Models

**DOI:** 10.3390/s22103650

**Published:** 2022-05-11

**Authors:** Pranesh Gopal, Amandine Gesta, Abolfazl Mohebbi

**Affiliations:** 1 Manipal Academy of Higher Education, Manipal 576104, India; pranesh.g1@learner.manipal.edu; 2 Department of Mechanical Engineering, Polytechnique Montréal, Montreal, QC H3T 1J4, Canada; amandine.gesta@polymtl.ca

**Keywords:** electromyography, gesture recognition, motion classification, assistive robots, deep learning, machine learning, prosthesis, transradial amputation

## Abstract

Upper limb amputation severely affects the quality of life and the activities of daily living of a person. In the last decade, many robotic hand prostheses have been developed which are controlled by using various sensing technologies such as artificial vision and tactile and surface electromyography (sEMG). If controlled properly, these prostheses can significantly improve the daily life of hand amputees by providing them with more autonomy in physical activities. However, despite the advancements in sensing technologies, as well as excellent mechanical capabilities of the prosthetic devices, their control is often limited and usually requires a long time for training and adaptation of the users. The myoelectric prostheses use signals from residual stump muscles to restore the function of the lost limbs seamlessly. However, the use of the sEMG signals in robotic as a user control signal is very complicated due to the presence of noise, and the need for heavy computational power. In this article, we developed motion intention classifiers for transradial (TR) amputees based on EMG data by implementing various machine learning and deep learning models. We benchmarked the performance of these classifiers based on overall generalization across various classes and we presented a systematic study on the impact of time domain features and pre-processing parameters on the performance of the classification models. Our results showed that Ensemble learning and deep learning algorithms outperformed other classical machine learning algorithms. Investigating the trend of varying sliding window on feature-based and non-feature-based classification model revealed interesting correlation with the level of amputation. The study also covered the analysis of performance of classifiers on amputation conditions since the history of amputation and conditions are different to each amputee. These results are vital for understanding the development of machine learning-based classifiers for assistive robotic applications.

## 1. Introduction

The upper limb, consisting of the arm, elbow, forearm, wrist, and fingers, is the most dexterous part of the human body that makes activities of daily living possible. The human hand has seven axes along with 27 degrees of freedom of wrist and fingers that helps in the hard and soft manipulation of objects in our dynamic environment [1]. Loss of even a part of it is detrimental for smooth movements in activities of daily living (ADL). As of 2005, close to half a million people have an upper limb amputation and every year, 50,000 new limb amputations are performed, inflating the number even further [2]. Upper limb amputations are performed due to several reasons, but 70% of total amputations are due to trauma-related accidents and the rest by non-contagious diseases such as cancer, congenital deformities etc. [3]. There are four types of upper limb amputations, namely wrist disarticulation (on the wrist), transradial (TR) amputation (below the elbow), transhumeral (TH) amputation (above the elbow and below the shoulder), and shoulder disarticulation (on shoulder). Each amputation affects the lives of the amputees to different extents, making their living partially dependent to fully dependent. Upper limb amputees can opt for various prosthetic solutions available on the market to make them independent to carry out activities of daily living. The prosthetic solution can either be passive or active based on its function [4]. There are body-powered prosthetic solutions that rely on another part of the body for actuation; usually, this comes with a limited degree of freedom and chaotic actuation [5]. On the other hand, myoelectric prosthetic solutions that rely on neuro-muscular electromyography for actuation is more intuitive, but they are yet to be made reliable and robust for more degrees of motion since bio-signals are very noisy. This paper is primarily about analyzing the back end of the myoelectric prosthesis comprising pattern recognition for appropriate motion actuation for transradial amputees. 

The major components of a myoelectric prosthetic system are signal acquisition, data processing, feature extraction, pattern recognition, and actuation. Even though most of these components are similar for any intelligent bio-signal based solutions, the differences amongst amputees who will use such a system pose additional challenges: in amputees, the absent muscle responsible for actuation and the corresponding phantom limb movement which varies across amputees. Hence, the control aspects in EMG-based robotic solutions may vary, which makes each component convoluted and challenging for amputees. Below, each system component is described briefly:

1. *Sensing and Data acquisition*: An active upper limb prosthesis relies on EMG electrodes, an inertial measurement unit (IMU), and an inclinometer for the acquisition of muscle signal, movement of arm, and tilt of the arm, respectively, for understanding the motion intention in the users. These sensors are sensitive to noise making it harder to extract relevant information. Furthermore, the placement of these sensors is not standardized since amputees have different kinds of stump and amputation. Furthermore, bio-signals acquired from amputees are noisier than intact counterparts due to the absent muscle. Thus, data acquisition of bio-signals is challenging, especially in amputees.
2. *Data Processing*: Since EMG signals obtained are polluted with motion artefacts, electronics interference, electro cardiac signals, ambient noise, etc., there is a need to clearly understand the signals and process them accordingly to make it fit for classification of motion intentions. This stage includes rectification, filtering, and normalization for removing the noise and increasing readability of the data acquired through the sensors.
3. *Feature extraction*: Features are the usable units of data from raw signals. Feature extraction is a fundamental component to transform the raw data into usable input for the classification algorithm. There are three kinds of features in EMG signals: time domain features, frequency domain features, and time-frequency domain.
  - *Time Domain Features*: These are features captured from the amplitude of an electric signal in each period. Root Means Square (RMS), Window Length (WL), Slope Sign Change (SSC), Zero Crossing (ZC), Enhanced Mean Absolute Value (EMAV), etc., are some of the time domain features used in relevant works [6,7].
  - *Frequency Domain Features*: These features are extracted by Fourier frequency decomposition. Short fast Fourier transform (SFFT) and discrete fast Fourier transforms (DFFT) of the signal are some of the features used in previous studies [8].
  - *Time-Frequency Features*: These features are extracted in the complex domain of time and frequency characteristics. Wavelet transforms are one of the features used in previous studies [9] Throughout this study, time domain features are considered since they are computationally less expensive compared to their counterparts.
4. *Motion Intention Classification*: The features extracted in the previous step are used to build a motion intention classifier. The conventional myoelectric systems utilize thresholding techniques for classification that is limited due to the variability in signals due to fatigue, change in placements of electrodes, and supporting fewer activity classes. In contrast, machine learning classifiers can learn from the data and capture the variability in the model. Different machine learning algorithms have been used for EMG-based activity classification such as Support Vector Machines (SVM), Linear Discriminant Analysis (LDA), Ensemble Learning, K-Nearest Neighbor (KNN), Random Forest, Artificial Neural Networks (ANN), etc. [10,11,12,13].
5. *Robotic actuation*: During this stage, the robot actuators will be activated according to the result of motion intention classification to perform the intended activity with the robotic or prosthetic arm. The motion intentions will translate into specific motion trajectories and applied torques/forces through a kinematic/dynamic model of the robotic arm.

Although the given components appear straightforward, there exist a couple of major limitations in the currently available approaches. Most of the research studies focus on EMG classification of activities in intact subjects and directly correlate to the possible success of their technique in amputees [14]. There is scarcity in well-maintained labelled EMG datasets consisting of amputees; hence, most of the best-performing methods are non-replicable and not reliable. Moreover, the majority of studies in multi-class EMG-based motion intention classification take the accuracy of the model as a benchmark in contrast to overall generalization exhibited by performance metrics such as the F1 score [15,16,17] 

Lastly, systemic studies of the impact of data processing in performance of the classification model are scarce and the field is still unexplored. Hence, our work sheds light on variations in performance on different data processing and modeling techniques for classifying movement intentions based on EMG sensor data of participating muscles. 

The major contributions of this paper are:

- Developing and comparing machine learning and deep learning models for classification of motion intentions for transradial (TR) amputees.
- Benchmarking the performance of the motion intention classifiers based on overall generalization across various classes.
- Systematic study on the impact of sliding window length and time domain features on the performance of classification models.

## 2. Relevant Works

Machine learning algorithms have been proven to be a powerful alternative for pattern recognition across multiple disciplines. It is reasonable to view machine learning as a possible means of prediction of motion intention using EMG signals. The earliest EMG classifier was based on SVM, later with the advent of exploration of other learning algorithms, many more algorithms such as Decision tree, KNN, and ANN were used in classification [10,13,18] 

The EMG signals data are modeled as either image (2D array) or time series or combination of both as input to classical machine and deep learning algorithms [4,5,6,7]. KNN, SVM, ANN, and random forest form time series feature-based models. Since the variability of the EMG data between subjects is high, appropriate classification algorithms should be used that are not highly sensitive to this variability. Recurrent Neural Networks (RNNs), Convolutional Neural Networks (CNNs), and Temporal Convolutional Networks (TCNs) form featureless classification models that do not involve feature extraction [19,20]. In our work, we explore both the categories of models for comparison.

Chen et al. [21] explored creating a more generalized deep neural network-based classifier called EMG net built with CNN that is compact, i.e., with fewer parameters compared to traditional CNN. The classification accuracy of EMG net is reported to be close to 93% using the NinaPro DB5 [22,23], where a transfer learning approach was used on pretrained deep learning models to generalize better and minimize training. The model had an accuracy of 97.81% across 17 intact subjects on seven wrist gestures. The paper focused mostly on the recognition for robotic control of 6 DOF robotic arm than on analyzing the model’s performance on amputees. 

It is interesting to note that Coté-Allard et al., Demir et al., and many other researchers used private databases that limited other researchers from reproducing or further exploring other methods to improve the performance of the classifiers [17,23]. NinaPro is the effort in the direction of creating a standardized public EMG dataset with commendable maintenance. (http://ninaweb.hevs.ch/, accessed on 1 September 2020). There are ten datasets of both intact and amputee’s data along with the data from different sensors [24]. The benchmarked accuracy is close to 30–50% in TR amputees [25]. M. Atzori et al. proposed a CNN model that recorded 38.09 ± 14.29% accuracy on the same database for 50 movements for all the subjects in the database [15]. 

In addition to the selection of the algorithm, sliding window size is also a significant contributor in determining performance of the model in both feature-based and non-feature-based learning. The constraint being 300 ms is maximum delay between intention and actuation of prosthetic solution for smooth and reliable control. A. Ullah et al. explored different sliding window sizes with various classifier algorithms. Furthermore, our work builds on different sliding window sizes and examination of its performance with different classifier algorithms across subjects [26].

Most of the research in machine learning favors average accuracy as a performance of interest [17,25,27,28]. The limitation with average accuracy is that it conveys close to nothing about the generalization of the model across different motion intention. Hence, our work adopts both average accuracy and average F1-score as performance indices for comparison of classifiers. 

## 3. Methodology

### 3.1. Dataset

The NinaPro dataset is used as the dataset as its one of the most reliable and well-maintained databases of EMG signals of both intact and amputees. The database serves as an open-source dataset for analysis and improvements in pattern recognition for well-rounded robotic prosthetic solutions for amputees. Since the primary objective of our study is on motion intention recognition of TR amputees through their EMG signals, Database 3 of NinaPro, which contains EMG signals of 11 TR amputees, was used. 

**Figure 1:**
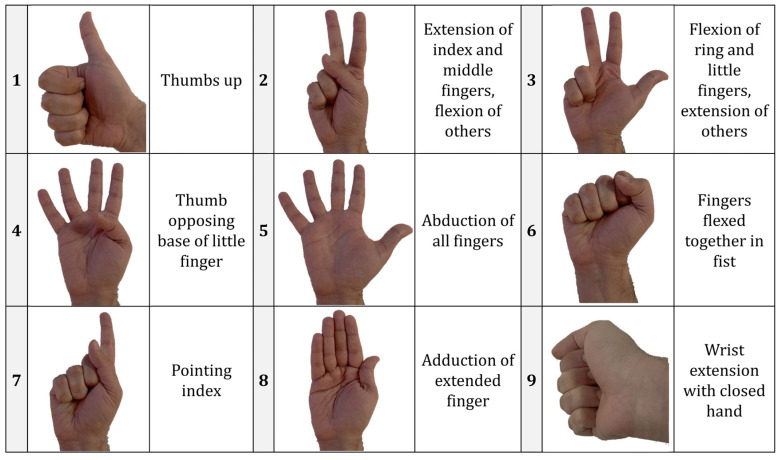
Gestures of interest used for the EMG classification based on the NinaPro dataset [25].

The dataset was acquired from transradial amputee subjects mimicking through their amputated arm, as far as possible, along with movies of the motion gestures from laptop. The database relies on the phantom limb activity of the amputees that are captured through 12 EMG electrodes placed uniformly around the region of amputation. Through guided visual stimuli, the subjects perform the 50 gestures comprising different finger movements, rotations, force, and grasps [24]. Apart from EMG signal, an inertial measurement unit (IMU), an inclinometer, and a force sensor are used to collect the data throughout the data acquisition process. The paper objectively analyzes the reliability of EMG signals alone for motion intention in amputees; hence, only the EMG signals alone were used to produce motion intention classification models. The EMG signals were acquired through delayed electrodes with a sampling frequency of 2 kHz and shielded to a 50 Hz power line, ensuring a less noisy signal. Out of 50 gestures given, 10 of the most common gestures that are close to impossible for the TR amputees were selected (Figure 1). The number of subjects was further shortened to four and care was taken to capture the variability of the degree of amputation and performance of the model. Table 1 summarizes the details of the amputee subjects’ data used in this research. 

### 3.2. Data Processing 

The raw EMG data must be processed to reduce the noise and extract relevant features for the classification algorithm. The details of the processing done in our work is described below.

#### 3.2.1. Preprocessing

Normalization was not required due to absence of maximum amplitude potential and since the data acquisition was performed in single session, it was not needed [14]. Second order Butterworth lowpass filter of the cutoff frequency of 1 Hz, 5 Hz, 50 Hz, 10 Hz, and bandpass filter of 50–450 Hz was used to compare which was the best cutoff frequency through the Support Vector Machine (SVM). These cutoff frequencies were selected from related literature. Low pass Butterworth filter with 1 Hz cutoff outperformed other filter design significantly. Moreover, rectification was performed to obtain the envelope of the signals. 

#### 3.2.2. Sliding Window and Feature Extraction

Different sliding window lengths of 50 ms, 100 ms, 150 ms, 200 ms, and 250 ms were used to arrive at optimum sliding window length with 50% overlap for better performance of the classifier. The study focuses on time-domain features since it is computationally less expensive and faster to compute translating to faster classification. The features used for the study are Root Mean Square (RMS), Enhanced Mean Absolute Value (EMAV), Waveform length (WL), and Variance (VAR). The mathematical representations of each of the features are described in Equations (1)–(4) as: 

Root Mean Square,

(1) $$\mathrm{RMS}=\sqrt{\frac{1}{N}\sum_{i}^{N}x_{i}^{2}}$$

Variance,

(2) $$\mathrm{VAR}=\frac{1}{N}\sum_{i-1}^{N}{\left(x_{i}-\mu \right)}^{2}$$

Enhanced Mean absolute Value,

(3) $$\mathrm{EMAV}=\frac{1}{N}\sum_{i=1}^{N}\left|{\left(x_{i}\right)}^{p}\right|,\;p=\{\begin{array}{c} 0.75,\;if\;i\geq 0.2N\&i\leq 0.8N\\ 0.50,\;otherwise \end{array}$$

Waveform Length,

(4) $$\mathrm{WL}=\sum_{i=1}^{N-1}\left|x_{i+1}-x_{i}\right|$$

where N is the number of samples used for calculation, $x_{i}$ is the *i*th sample of measurement, and μ is the average value of x. The classifiers were built upon using individual and a combination of time domain features to select the optimum feature resulting in higher performance (Table 2).

### 3.3. Classification Algorithm

To analyze the performance of the model across different algorithms and different subjects with transradial amputation, six machine learning/deep learning algorithms were used to build the classifier: Tree algorithm, K-Nearest Neighbor (KNN), Support Vector Machine (SVM), Linear Discriminant Analysis (LDA), Ensemble learning, Artificial Neural Network (ANN), and Convolutional Neural Network (CNN). The learning models of this study were selected based on the original classification case study of NinaPro dataset published in [24,29]. Moreover, the most common classification models surveyed in [28,30] have been considered in this work. Except for CNN, all the other algorithms are trained on extracted features. The implementation and parameter tuning of classical machine learning models was carried out using MATLAB scripting, and the TensorFlow framework was used for building deep learning classifiers using Python. The training of all the models was performed in the Cedar cluster belonging to Compute Canada (CC).

#### 3.3.1. Feature-Based Learning

**Figure 2:**
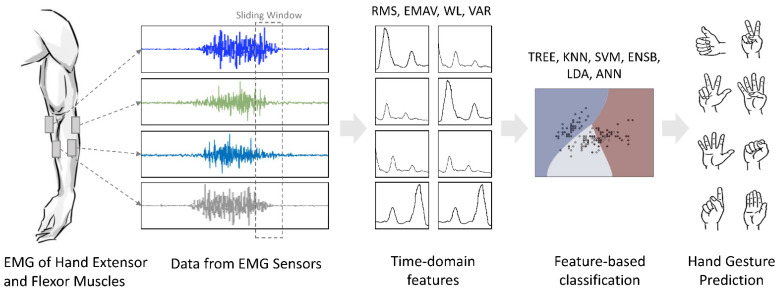
Feature-based learning for classification of hand gestures using NinaPro dataset.

Each of above-mentioned classical machine learning and ANNs were trained on the features extracted from various sliding window lengths and overlaps. This process of feature-based learning is illustrated in Figure 2. 

Various combinations of the features were used to train each algorithm to verify the performance of each of the selected algorithms. The performance metrics for the study were average accuracy and average F_1_ score as they elucidated both accuracy and overall learning of each category of classification. These metrics were calculated as:

(5) $$\mathrm{Accuracy}=\frac{\left(\mathrm{TP}+\mathrm{TN}\right)}{\left(\mathrm{TP}+\mathrm{FP}+\mathrm{TN}+\mathrm{FN}\right)}$$

(6) $$F_{1}\;\mathrm{Score}=\frac{\mathrm{TP}}{\mathrm{TP}+\frac{1}{2}\left(\mathrm{FP}+\mathrm{FN}\right)}$$

where TP is the total number of true positive, FP is the total number of false positive, TN, is the total number of true negative, and FN is the total number of false negative recognitions. To analyze the generalized learning and reliability of the model, 10-fold cross validation and careful monitoring of loss function was done. The hyperparameter tuning was performed by Keras Tuner on each of the classifiers to boost its performance. 

#### 3.3.2. Non-Feature-Based Learning

**Figure 3:**
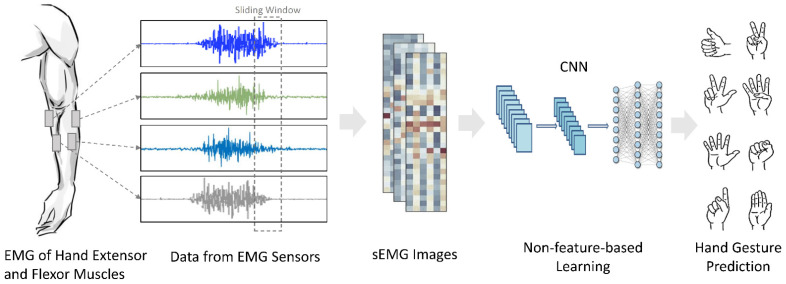
Non-feature-based learning using a Convolutional Neural Network (CNN) for hand gesture classification using NinaPro dataset.

Convolutional Neural Network is the algorithm used as a non-feature-based learning classifier. The dataset was augmented such that each sliding window (2D matrix input) of the EMG signals was fed to the algorithm. The EMG data were treated as image and the kernels slid over the image to learn more generalized features. This non-feature-based learning approach is described in Figure 3.

**Figure 4:**
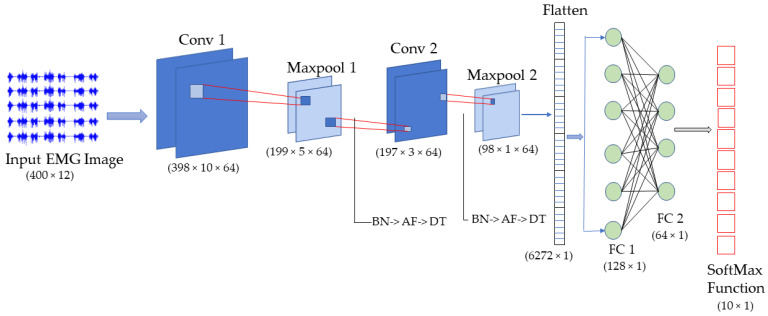
The architecture of the proposed CNN model with input image as obtained from 200 ms sliding window length used in the study. The hyperparameters, such as learning rate, neurons in fully connected layers, and filter size were tuned during hyperparameter tuning in each of the model.

CNNs are known for capturing features from the dataset. This is made possible by a convolutional layer where the kernel smaller than the image captures meaningful information and the pooling layer provides the statistical summary of the information, making them computationally lighter. Nonlinearity is introduced to the network through the activation function. Since CNNs are known to overfit easily because of millions of parameters encompassed within it, dropouts and batch normalization can be essential to prevent this and generalize the model well. The proposed CNN was built with two convolutional layers followed by a Maxpool layer sandwiched with dropouts (DT), tanh activation function (AF), and batch normalization (BN). The end result was two Fully Connected (FC) layers with dropout in between leading to SoftMax layer (Figure 4). The hyperparameters were tuned for each of the models with Keras Tuner. 

The performance indexes used were accuracy and F1 score. There was appropriate 10-fold cross-validation and observation of loss function and accuracy on each mini batch that is trained. The learning rate used is 0.001 and the Adam optimizer [31].

Both categories of models were trained across all selected subjects several times and their average performance was recorded. Moreover, several input sliding window lengths and overlaps were experimented with to analyze their impact on the performance of the classifier. 

## 4. Results

### 4.1. Classification Algorithms and Their Performance

**Figure 5:**
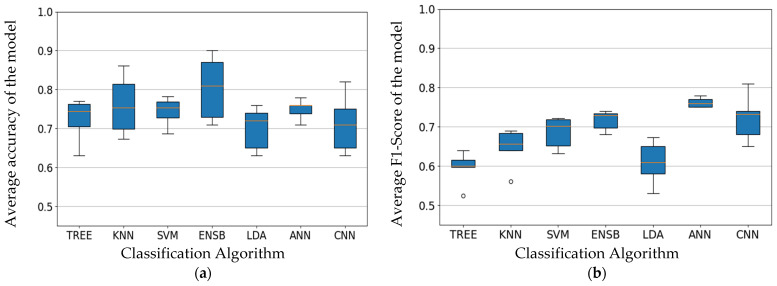
(**a**) Average accuracy for models trained for all subjects and sliding window across different classification algorithms. (**b**) Average F1-score for models trained for all subjects and sliding window across different classification algorithms. Orange line refers to the median performance metric and circles depict the outliers in the distribution.

The performance metrics were calculated for each of the machine learning models (Tree, KNN, SVM, LDA, ANN, Ensemble, CNN) trained on five different sliding window lengths (50 ms, 100 ms, 150 ms, 200 ms, 250 ms; 50% overlap) across all subjects. The maximum accuracy of each of the algorithm on each sliding window length was analyzed for comparison. It was observed that the accuracy obtained by the dataset is far better than the benchmark metric in Atzori et al. work. This can be attributed to the narrowed activity of interest and hyperparameter optimization. The accuracy of CNN and Ensemble learning-based classifiers were investigated and Ensemble had higher median accuracy than all the algorithms. The accuracy has always been the standard for the performance of EMG-based activity classification. It often leads to an accuracy trap. The average accuracy of the model has failed to convey the overall generalization across all the classes which are crucial for reliable classification. Figure 5a,b show the average accuracy and F1 score across different algorithms to analyze the difference in trend between average accuracy and F1 score. Plots demonstrate that the most accurate ensemble model does do not perform well when compared to that based on F1 scores. The deep learning algorithms performed better in terms of generalization-based metrics. The classification algorithms of significance were an Ensemble, SVM, ANN, and CNN, which performed better than the rest based on accuracy, F1 score, and variance in the boxplot. 

### 4.2. Performance of Subject

**Figure 6:**
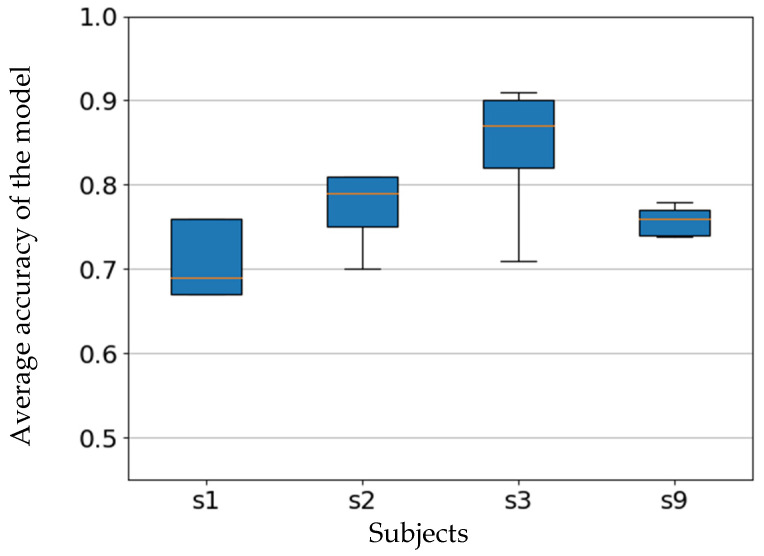
Boxplot of accuracy across different subjects (s1, s2, s3, s9) on top four classification algorithms (CNN, ANN, Ensemble, SVM).

The ANN, CNN, SVM, and Ensemble algorithm, as the applied classification algorithms being the top-four performing models, had varied results on each of the subjects. Figure 6 was plotted with the highest accuracy among the four algorithms on each of the subjects to understand the spread of performance that can explain the effect of the condition of amputation on the performance of the model. Subject three, having the least amount of remaining forearm, had the greatest spread in performance. Interestingly, deep learning algorithms perform the best in subjects with less remaining forearm than other classical machine learning algorithms and generalize better. Subjects 9 and 2, having the majority of the forearm (70%, 90%), performed better and had lesser variance across the models compared to the other subjects. Subject 1 and subject 3 were more sensitive to changes in classification and data processing, making ANN and ensemble learning the best classification in most cases accommodating for varied amputation conditions. 

### 4.3. Performance of Sliding Windows

The sliding windows studied to analyze the relationship between performance of the classifiers and sliding window size for feature extraction-based learning models and featureless models were 50 ms, 100 ms, 150 ms, 200 ms, and 250 ms with 50% overlap. Windows of 300 ms and above were not considered since anything beyond 300 ms along with classification time would impact the real-time behavior of the assistive solutions [3]. The ANN model in feature-based learning model and CNN model in non-feature-based learning model were studied across five sliding window sizes to compare between the approaches of learning models. 

**Figure 7:**
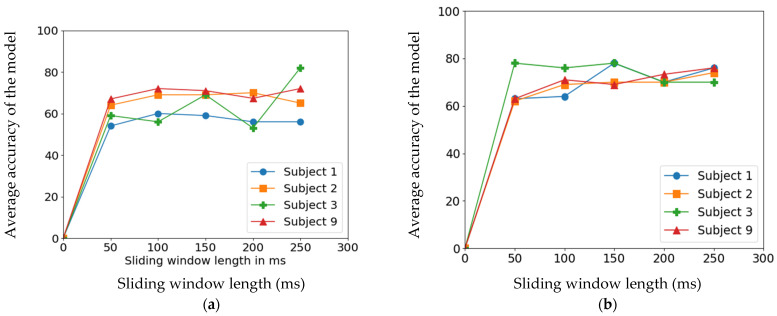
Classification accuracy on various sliding window lengths with 50% overlap on ANN algorithm across subjects. (**a**) represents the trend of accuracy on varying sliding windows in the ANN-based classifier and (**b**) represents the trend of accuracy on varying sliding window lengths in the CNN-based classifier.

Figure 7 clearly shows that the featured-based classification algorithm performed better on increasing the sliding window length. In contrast, featureless CNN classification algorithm performed steadily across the varying sliding window. The peculiar behavior of subject 3 in both plots can be an indication that in more severe amputation, choice of sliding windows had a significant effect on model performance. 

### 4.4. Performance on Features

Selection of feature is one of the crucial steps in achieving a good performance of the classifier. The performance of our classifier depends on time domain features which are computationally less expensive compared to frequency domain features. Since the causal nature of the signal is being used, selection of the right feature matter even more. The study on features revealed that a combination of features (Q5 combination of all features as given in Table 2) yielded the best results in all the subjects with various algorithms. Even though combination features yielded the best performance, it was at the cost of dimensionality increase and computational complexity. Next, the best single feature was Root Mean Square (RMS-Q1) that performed consistently well throughout all subjects and all algorithms (refer to Table 3). To verify whether this trend was corelated with sliding window size, the classifier was built for each of the window sizes as mentioned in the previous section. The result confirmed the trend with some spikes in performance of variance and waveform length (WL-Q3 and VAR-Q4), performing at par or better than RMS. Hence, the combination of features and RMS performed better than any other features in the majority of cases irrespective of sliding window size and overlap.

## 5. Conclusions

The aim of the study was to understand the relationship between feature extraction and sliding window length across different classification algorithms with reference to the accuracy and F1 score as a more rounded approach on analyzing the performance. Ensemble learning and deep learning algorithms performed better than other classical machine learning algorithms such as SVM, KNN, LDA, and Tree. Investigating the trend of varying sliding window on feature-based and non-feature-based classification models revealed an interesting correlation with the level of amputation. The study also covered the analysis of performance of classifiers on amputation conditions since the history of amputation and conditions are different to each amputee. These results are vital for understanding the development of machine learning-based classifiers for assistive robotic applications. 

## 6. Future Work

Learning-based classifiers possess a huge potential in motion intentions classification and development of the assistive robotic systems based on them for amputees. The major limitation of any machine learning and deep learning models is the requirement of huge datasets to build cutting edge, classification models. The dataset used for the research comprises very few subjects and the quantity of data is limited; any generalization on the study of EMG signals needs a bigger, well-maintained dataset. The study undertaken here can serve as the backbone for further analysis on the effect of preprocessing on the dataset over the generalization of classifiers both within and across different subjects, to ensure the robustness and reliability of EMG based amputee solutions. Furthermore, a detailed study on computation can be performed for achieving real time classification for the reliability of solutions. Various assistive systems can be built over the classification model to further uplift subjects’ lives and make them independently able to carry out their activities of daily living.

## Figures and Tables

**Table 1:** Details of the transradial amputee subjects relevant to the research [25].

Subject	Handedness	Amputated Hand	Remaining Forearm (%)	Years since Amputation	Phantom Limb Sensation	DASH Score	Number of Electrodes Used
1	R	R	50	13	2	1.67	12
2	R	L	70	6	5	15.18	12
3	R	R	30	5	2	22.5	12
9	R	R	90	14	5	3.33	12
Average Score			60	9.5	3.5	10.67	

**Table 2:** Definition of classifiers based on the features used.

Classifier	Features Combination
Q1	RMS
Q2	WMAV
Q3	WL
Q4	VAR
Q5	RMS + EMAV + WL + VAR

**Table 3:** Accuracy of different combinations across different subjects using.

Ensemble Classifier Accuracy (200 ms)	Subject 1	Subject 2	Subject 3	Subject 9
Q1	60.32%	65.53%	71.60%	66.90%
Q2	60.81%	63.59%	71.25%	65.50%
Q3	61.50%	62.28%	70.90%	69.17%
Q4	59.50%	63.75%	70.87%	69.03%
Q5	63.13%	65.57%	75.37%	70.70%

## Data Availability

The data presented in this study are available on request from the corresponding author. The data are not publicly available owing to continuing study by the authors.

## References

[1] Elkoura G., Singh K. Handrix: Animating the Human Hand. Proceedings of the 2003 ACM SIGGRAPH/Eurographics Symposium on Computer Animation.

[2] Ziegler-Graham K., MacKenzie E.J., Ephraim P.L., Travison T.G., Brookmeyer R. (2008). Estimating the Prevalence of Limb Loss in the United States: 2005 to 2050. Arch. Phys. Med. Rehabil..

[3] Englehart K., Hudgins B. (2003). A Robust, Real-Time Control Scheme for Multifunction Myoelectric Control. IEEE Trans. Biomed. Eng..

[4] Maat B., Smit G., Plettenburg D., Breedveld P. (2018). Passive Prosthetic Hands and Tools: A Literature Review. Prosthet. Orthot. Int..

[5] Millstein S.G., Heger H., Hunter G.A. (1986). Prosthetic Use in Adult Upper Limb Amputees: A Comparison of the Body Powered and Electrically Powered Prostheses. Prosthet. Orthot. Int..

[6] Tkach D., Huang H., Kuiken T.A. (2010). Study of Stability of Time-Domain Features for Electromyographic Pattern Recognition. J. NeuroEng. Rehabil..

[7] Samuel O.W., Zhou H., Li X., Wang H., Zhang H., Sangaiah A.K., Li G. (2018). Pattern Recognition of Electromyography Signals Based on Novel Time Domain Features for Amputees’ Limb Motion Classification. Comput. Electr. Eng..

[8] Phinyomark A., Khushaba R.N., Scheme E. (2018). Feature Extraction and Selection for Myoelectric Control Based on Wearable EMG Sensors. Sensor.

[9] Tuncer T., Dogan S., Subasi A. (2020). Surface EMG Signal Classification Using Ternary Pattern and Discrete Wavelet Transform Based Feature Extraction for Hand Movement Recognition. Biomed. Signal Process. Control.

[10] Toledo-Pérez D.C., Rodríguez-Reséndiz J., Gómez-Loenzo R.A., Jauregui-Correa J.C. (2019). Support Vector Machine-Based EMG Signal Classification Techniques: A Review. Appl. Sci..

[11] Yaman E., Subasi A. (2019). Comparison of Bagging and Boosting Ensemble Machine Learning Methods for Automated EMG Signal Classification. BioMed Res. Int..

[12] Dellacasa Bellingegni A., Gruppioni E., Colazzo G., Davalli A., Sacchetti R., Guglielmelli E., Zollo L. (2017). NLR, MLP, SVM, and LDA: A Comparative Analysis on EMG Data from People with Trans-Radial Amputation. J. NeuroEng. Rehabil..

[13] Gokgoz E., Subasi A. (2015). Comparison of Decision Tree Algorithms for EMG Signal Classification Using DWT. Biomed. Signal Process. Control.

[14] Peerdeman B., Boere D., Witteveen H., in ‘t Veld R.H., Hermens H., Stramigioli S., Rietman H., Veltink P., Misra S. (2011). Myoelectric Forearm Prostheses: State of the Art from a User-Centered. J. Rehabil. Res. Dev..

[15] Atzori M., Cognolato M., Müller H. (2016). Deep Learning with Convolutional Neural Networks Applied to Electromyography Data: A Resource for the Classification of Movements for Prosthetic Hands. Front. Neurorobot..

[16] Barron O., Raison M., Gaudet G., Achiche S. (2020). Recurrent Neural Network for Electromyographic Gesture Recognition in Transhumeral Amputees. Appl. Soft Comput. J..

[17] Côté-Allard U., Fall C.L., Drouin A., Campeau-Lecours A., Gosselin C., Glette K., Laviolette F., Gosselin B. (2018). Deep Learning for Electromyographic Hand Gesture Signal Classification Using Transfer Learning. IEEE Trans. Neural Syst. Rehabil. Eng..

[18] Mattioli F.E., Lamounier E.A., Cardoso A., Soares A.B., Andrade A.O. Classification of EMG Signals Using Artificial Neural Networks for virtual Hand Prosthesis Control. Proceedings of the 2011 Annual International Conference of the IEEE Engineering in Medicine and Biology Society.

[19] Shen S., Gu K., Chen X.R., Yang M., Wang R.C. (2019). Movements Classification of Multi-Channel SEMG Based on CNN and Stacking Ensemble Learning. IEEE Access.

[20] Zanghieri M., Benatti S., Burrello A., Kartsch V., Conti F., Benini L. (2020). Robust Real-Time Embedded EMG Recognition Framework Using Temporal Convolutional Networks on a Multicore IoT Processor. IEEE Trans. Biomed. Circuits Syst..

[21] Chen L., Fu J., Wu Y., Li H., Zheng B. (2020). Hand Gesture Recognition Using Compact CNN via Surface Electromyography Signals. Sensor.

[22] Côté-Allard U., Fall C.L., Campeau-Lecours A., Gosselin C., Laviolette F., Gosselin B. Transfer Learning for SEMG Hand Gestures Using Convolutional Neural Networks. Proceedings of the IEEE International Conference on Systems, Man, and Cybernetics (SMC).

[23] Demir F., Bajaj V., Ince M.C., Taran S., Şengür A. (2019). Surface EMG Signals and Deep Transfer Learning-Based Physical Action Classification. Neural Comput. Appl..

[24] Atzori M., Gijsberts A., Heynen S., Hager A.-G.M., Deriaz O., van der Smagt P., Castellini C., Caputo B., Müller H. Building the NINAPRO Database: A Resource for the Biorobotics Community. Proceedings of the IEEE RAS and EMBS International Conference on Biomedical Robotics and Biomechatronics.

[25] Atzori M., Gijsberts A., Castellini C., Caputo B., Hager A.G.M., Elsig S., Giatsidis G., Bassetto F., Müller H. (2014). Electromyography Data for Non-Invasive Naturally-Controlled Robotic Hand Prostheses. Sci. Data.

[26] Ullah A., Ali S., Khan I., Khan M.A., Faizullah S. (2020). Effect of Analysis Window and Feature Selection on Classification of Hand Movements Using EMG Signal. Intelligent Systems and Applications.

[27] Li G., Kuiken T.A. EMG Pattern Recognition Control of Multifunctional Prostheses by Transradial Amputees. Proceedings of the 2009 Annual International Conference of the IEEE Engineering in Medicine and Biology Society.

[28] Geng W., Du Y., Jin W., Wei W., Hu Y., Li J. (2016). Gesture Recognition by Instantaneous Surface EMG Images. Sci. Rep..

[29] Atzori M., Müller H. The Ninapro Database: A Resource for SEMG Naturally Controlled Hand Prosthetics. Proceedings of the Annual International Conference of the IEEE Engineering in Medicine and Biology Society.

[30] Inam S., Al Harmain S., Shafique S., Afzal M., Rabail A., Amin F., Waqar M. (2021). A Brief Review of Strategies Used for EMG Signal Classification. Proceedings of the 2021 International Conference on Artificial Intelligence, ICAI 2021.

[31] Kingma D.P., Ba J. Adam: A Method for Stochastic Optimization. Proceedings of the International Conference on Learning Representations ER.

